# Analyses of the roles and potential targets of m7G-related genes in colorectal cancer using single-cell and bulk RNA sequencing data

**DOI:** 10.1371/journal.pone.0337288

**Published:** 2025-12-17

**Authors:** Jie Hu, Zhihua Chen, Chenyang Ma, Yuan Fang, Quanfa Li, Jiebin Zheng, Jiannan He, Suyong Lin, Shaoqin Chen

**Affiliations:** Department of Gastrointestinal Surgery, Mengchao hepatobiliary hospital of Fujian Medical University, Fuzhou, Fujian, China; Mohammed VI Polytechnic University: Universite Mohammed VI Polytechnique, MOROCCO

## Abstract

**Background:**

The pathogenesis of colorectal cancer is complex and difficult to treat, and there is a risk of metastasis and recurrence. m7G modification as a kind of RNA modification has been widely concerned in the field of tumor. However, there are few research in the field of CRC. This study aims to elucidate the effects of m7G modification on CRC from the perspective of single cell transcriptome and search for potential therapeutic targets.

**Methods:**

We downloaded the single cell dataset using the GEO database and processed the data using the Seurat R package. gene ontology (GO) and Kyoto Encyclopedia of Genes and Genomes (KEGG) were used to analyze differential genes. CellChat is used for cell communication analysis. NMF differentiates subtypes. CIBERSORT and xcell are used to analyze immune cells. Lasso COX regression was used to search for hub genes. We explored the biological functions of NUDT10 utilizing biological experiments.

**Results:**

Most m7G-related modification genes were significantly higher in expression in tumor tissues compared to normal tissues, primarily within epithelial cells. The DEGs of m7G-related genes expressed were mainly enriched in inflammation and metabolic pathways. NCBP2 and EIF3D could promote the occurrence and development of malignant epithelial tumor cells. In cellchat, m7G-related genes high group showed stronger interactions. m7G also affects tumor immune microenvironment and metabolism. In addition, seven genes were chosen for prognostic model construction. Biological experiments have demonstrated that NUDT10 promotes CRC progression.

**Conclusion:**

This research revealed tumor growth and microenvironment changes mediated by m7G modification and constructed a prognostic model based on the hub genes, which will guide further exploration of m7G modification.

## Introduction

The incidence and mortality rates of colorectal cancer (CRC) are extremely high around the world. According to global cancer statistics for 2020, there are over 1.9 million new cases of CRC each year, with approximately 935,000 deaths [1,2]. In developed countries, there is a decreasing trend in the incidence due to early endoscopic detection and changes in risk factors [3]. However, as the population ages, the incidence of CRC and its economic burden increase [4,5]. In recent years, treatment for CRC has primarily involved surgery and chemotherapy, but there are still problems with recurrence, resistance, and metastasis [6,7]. Researchers continue to gain insights into CRC, with targeted therapy and immunotherapy becoming new options. For certain patient subtypes, the options include monoclonal antibody therapy targeting EGFR (cetuximab and panitumumab) and VEGF (bevacizumab) in combination with chemotherapy [8,9]. Deficiencies in mismatch repair and high microsatellite instability (d-MMR/MSI-H) account for approximately 15% of all CRCs, and only immunotherapy is effective in these patients. However, there are issues related to drug resistance and recurrence [10]. The immune microenvironment, which includes macrophages, B cells, T cells, and neutrophils, contributes to the development and progression of tumors. There are three basic immune phenotypes of tumors: immune-inflamed, immune-excluded, and immune-deserted. Most CRCs are considered “cold” tumors with little T cell infiltration, existing in an immunosuppressive microenvironment, and transforming them into “hot” tumors with high immunogenicity remains a significant challenge [11,12]. Therefore, addressing the various challenges in CRC treatment requires molecular-level analysis of genomics, transcriptomics, and other aspects of CRC, as well as offering personalized treatment plans for tumor patients and changing the tumor immune suppression microenvironment to improve CRC prognosis.

Epigenetics refers to the stable inheritance of traits that occur without changes in the DNA sequence [13,14]. In eukaryotes, RNA, DNA, and histones undergo chemical modifications. Compared with studies on DNA and proteins, RNA research is less frequent. There are more than 170 types of chemical modifications of cellular RNA, including messenger RNA (mRNA), ribosomal RNA (rRNA), transfer RNA (tRNA), and small nuclear RNA (snRNA) [15–19]. RNA modifications affect RNA splicing, transcription, localization, and more [20]. Modifications such as N6-methyladenosine (m6A), N1-methyladenosine (m1A), 5-methylcytidine (m5C), N7-methyl-guanosine (m7G), and 2′-O-methylation (2′-O-Me) have been shown to be related to tumor development and progression [21]. Distinct from the m6A methyladenosine modification, m7G is another positively charged modification known as methylguanosine, which is commonly found at the 5’ end of tRNA, rRNA, and mRNA [22,23]. m7G has also been found to be involved in tumor immune regulation and drug resistance [24,25]. The m7G modification can stabilize tRNA and has an important impact on its expression and function, thereby improving mRNA translation efficiency. The m7G modification protects the mRNA from degradation outside the nucleus and regulates pre-mRNA splicing and mRNA translation [26,27]. In esophageal squamous cell carcinoma, the widely studied METTL1-WDR4 complex has been shown to modulate tRNA, thereby activating RPTOR and promoting ULK1 phosphorylation to inhibit autophagy [28]. METTL1 can also alter the structure of the pri-miRNA let-7e, inducing and maintaining the maturation of miRNA [29]. Chen et al. discovered that the interaction between QKI and its target mRNAs depends on the internal m7G modification mediated by METTL1, which, under stress conditions, affects the stability and translation efficiency of key genes in the Hippo signaling pathway [30]. METTL1-mediated m7G modification of tRNA promotes transformation into cancer by enhancing the translation of oncogene mRNAs [31]. Other molecules mediated by m7G modification in tumor regulation are less studied and require extensive experimental evidence to elucidate their mechanisms.

Although m7G has been reported to be associated with various cancers, such as lung, prostate, bladder, and CRC [32–34], the role of m7G-related regulators in CRC remains unclear. Therefore, elucidating their role and identifying potential therapeutic targets are of great significance. In this work, we evaluated the effect of m7G-related regulators on various CRC cell subgroups from a single-cell perspective. Using publicly available databases, we determined the distribution of m7G-related regulators in various cells and their effect on the tumor immune microenvironment. In The Cancer Genome Atlas (TCGA) database, we classified different patient characteristics based on m7G-related regulators, observed their different immune microenvironment characteristics, and elucidated the potential effects of m7G-related regulators on CRC from the perspective of the immune microenvironment. Finally, key molecules were selected to construct a prognostic model, and their roles were verified in a validation set. This study may guide future research on m7G.

## Methods

### Data Acquisition and Processing

Serial matrix data files for CRC and rectal cancer were obtained from the TCGA website. TCGA-COAD contains 498 samples, including 456 tumor samples and 42 normal tissue samples. TCGA-READ contains 175 samples, including 164 tumor samples and 11 normal samples. Conversion was performed based on official gene IDs and counts, and TPM data were extracted for subsequent analyses. The TPM underwent log2 processing, and all TCGA clinical data were downloaded from UCSC Xena. Samples with a survival time of 0 were removed, and 591 samples with prognostic information were used for the survival analysis. Gene Expression Omnibus (GEO) data were obtained from the GSE39582 CRC dataset (585 tumor tissue samples and 19 non-tumor samples) and GSE17536 (177 tumor tissue) [35]. GEO data were annotated using the GPL570 chip. Samples with a survival time of 0 were removed, and 562 samples with prognostic information were used for survival analysis.

This study used single-cell data from the GEO website’s GSE132465 dataset, which contained 67,296 cells, including 23 samples of CRC tumor tissue and 10 paired normal mucosa samples.

### Single-Cell Data Quality Control and Cell Annotation

The Seurat R package (Seurat version 5.0.0) was used to process the 10x Genomics raw data [36]. We excluded cells expressing mitochondrial genes more than 20%. Cells were filtered based on the criteria nFeature_RNA > 200 and nFeature_RNA < 600. Log normalization was used for data normalization. For single-cell dataset study, we employed the Harmony R package for batch effect correction, followed by downstream uniform manifold approximation and projection (UMAP) visualization and clustering analysis. Twenty principal components were used to generate cell clusters. Cell clustering was performed using the FindClusters function (resolution = 0.01). The top 10 differentially expressed genes were selected for cell annotation. Highly variable genes were annotated using literature and databases such as CellMarker and PanglaoDB [37].

### Choosing m7G-related Genes

Based on the gene set enrichment analysis database for m7G-related gene sets [38] and previously published articles [32,39,40], a total of 26 related genes(AGO, CYFIP1, DCP2, DCPS, EIF3D, EIF4A1, EIF4E, EIF4E2, EIF4E3, EIF4G3, GEMIN5, IFIT5, LARP1, LSM1, METTL1, NCBP1, NCBP2, NCBP3, NSUN2, NUDT10, NUDT11, NUDT16, NUDT3, NUDT4, SNUPN, WDR4) were selected for analysis.

**m7G-related Gene Scoring**

Gene set scoring was performed using the AUCell package in R. Scores were projected onto dot plots using t-SNE to determine which subgroups had higher scoring coefficients, with darker colors indicating higher gene set expression levels.

### Differential Analysis

Based on the median AUCell score, cells were grouped into high and low scores, and FindMarkers was used to identify genes with differential expression (|avg_log2FC| > 0.5 and Adjusted P-value (FDR) < 0.05.) for differential analysis. After converting the gene IDs, gene ontology (GO) and Kyoto Encyclopedia of Genes and Genomes (KEGG) pathway analyses were conducted to analyze genes with mutations, revealing the biological functions and signaling pathways involved in disease development and progression [41].

### Cell Communication Analysis

Based on the median score for the cell communication analysis, patients were divided into high- and low-score groups [42]. All data analysis and visualization were performed using CellChat for the high- and low-score groups.

### Pseudo-time Analysis

The Monocle 3 R package was used to conduct a pseudo-time analysis. Malignant epithelial cells were dimensionally reduced using UMAP, and ordered cells were used to specify the starting point for pseudo-time analysis. Finally, the effect of m7G-related gene expression on the development of malignant epithelial cells was analyzed.

### Metabolic Scoring

The scMetabolism R package was used for the analysis and visualization of metabolic pathway activity, available from (https://github.com/chenhao-qilu/scMetabolism/blob/main/R/compute_metabolism_Seurat.R) [43].

### NMF Clustering Analysis

The NMF package was used to determine m7G-related molecular subtypes from 591 CRC samples from the TCGA database [44]. The number of clusters, k, was set from 2 to 10, with the most appropriate k value selected for the subsequent cluster comparison analysis.

### Immune Infiltration and Correlation Analysis

CIBERSORT was used to analyze the immune microenvironment in 591 samples and observe differences in immune infiltration across different clusters [45]. Immune infiltration analysis of all samples was conducted using the xCell package, and the correlation between m7G-related genes and 68 types of immune cells was calculated. Correlations showing significant differences were displayed.

### Prognostic Risk Model Establishment and Validation

The least absolute shrinkage and selection operator (LASSO) Cox regression method was used to determine the ideal penalty parameter lambda and related coefficients based on the minimum criterion. Thirteen key genes were identified, and seven significant genes were selected to structure the prognostic model. Using the survival R package, Kaplan-Meier survival curves were analyzed using differential analysis of high- and low-score groups based on the median score. AUC scoring of the prognostic model was conducted using the PROC R package.

### RNA Sequencing (RNA-Seq) and Immunohistochemistry (IHC)

Fresh tissues (n = 22 per group) stored at Servicebio® RNA solid Tissue RNA Stabilization and Preservation Solution. RNA samples were submitted to **Igenebook** Co., Ltd. (wuhan, China) for library preparation and sequencing. This study was approved by the Ethics Committee of the First Affiliated Hospital of Fujian Medical University (Approval No. MRCTA, ECFAH of FMU [2023]572). Tissue samples were fixed in 4% paraformaldehyde and submitted to Sevier Biotechnology Co., Ltd. (Wuhan, China) for immunohistochemical (IHC) staining and scoring analysis.

### Cell transfection

The NUDT10 siRNA sequence was purchased from General Biol, and transfection was performed using Lipo8000 (Beyotime) according to the manufacturer’s protocol, followed by qPCR assay after 48 hours.

### RNA extraction and RT-qPCR

Following total RNA isolation with a commercial kit (Novozymes), first-strand cDNA was synthesized using Takara reverse transcriptase. Quantitative PCR was then performed on a QuantStudio 5 Flex instrument, where amplification was monitored in real-time using the intercalating dye SYBR Green I.

### Cell proliferation, apoptosis and migration assay

Following transfection, the cells were uniformly plated into 6-well plates. Cell proliferation was evaluated by utilizing a commercial EdU assay kit (Beyotime) according to the manufacturer’s instructions. Apoptosis was quantified using an Annexin V-APC/PI apoptosis detection kit (APE&Bio). The analysis for both proliferation and apoptosis was subsequently performed via flow cytometry on a CytoFLEX system (Beckman Coulter). To evaluate cell migration, cells were seeded into 6-well plates and cultured until reaching near 100% confluence. A uniform scratch was generated in the monolayer using a sterile 10 μL pipette tip. Images of the scratch were captured at 0 h and 24 h using a Leica inverted microscope. Migration rate (%) = [1 – (wound area at 24h/ wound area at 0h)] × 100%.

### Statistical analysis

R (version 4.3.2) was used to perform the statistics. For comparisons between normally distributed groups, we used the t-test, while for non-normally distributed groups, we used the Wilcoxon test. ANOVA was used for comparisons between three groups, and Pearson’s test was used for correlation.

## Results

### Overview of single-cell transcriptomics in CRC

**Fig 1:**
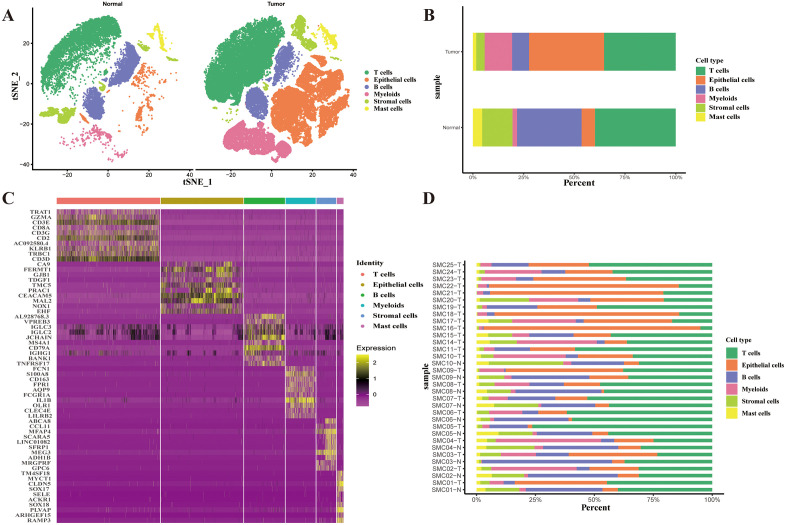
Overview of Single-Cell Transcriptomics in Colorectal Cancer. **A)** t-SNE plot of six cell clusters. **B)** Cell proportion in tumor and normal tissues. **C)** Heatmap of the top 10 genes in each cell cluster. **D)** Cell proportion in all samples.

Single-cell data from this study were sourced from the GEO dataset GSE132465, which comprises 33 single-cell sequencing samples, including 23 from patients with CRC and 10 from matched normal mucosal samples, totaling 65,362 cells. To advance our subsequent analyses, we used the Seurat software package for cell clustering and dimensionality reduction. All cells were dimensionally reduced using principal component analysis, followed by cell screening and quality control to remove unqualified cells (nFeature_RNA > 200, nFeature_RNA < 6000, and percent.mt < 20). Subsequently, t-SNE was used to divide single-cell data into six subgroups based on the reported classical markers: T cells, epithelial cells, B cells, myeloid cells, stromal cells, and mast cells (Fig 1A, S1 Fig). In Fig 1B, Tumor tissues and normal tissues had significantly different compositions of single cells, with a rise in the proportion of epithelial and myeloid cells and a notable decrease in T and B cells in tumor tissues, indicating an immunosuppressive state. Furthermore, we annotated the single-cell subgroups in each patient sample to observe the composition ratio of the cell subgroups per patient (Fig 1D). In tissue samples from different patients, T cells and epithelial cells were dominant, followed by B cells and myeloid cells. Samples sourced from normal tissues of the same patient had fewer T cells and epithelial cells than those from tumor tissues, except for patients SMC03 and SMC04, whose normal tissues had more T cells than the tumor tissues. Through Fig 1D, we discovered significant heterogeneity in the proportion of T cells per patient, which could either increase or decrease; however, overall, tumor tissues contained a higher number of epithelial cells than normal tissues. As markers, we also plotted the top 10 differentially expressed genes by cell type on a heatmap, as shown in Fig 1C.

### m7G-related gene expression in single-cell samples

**Fig 2:**
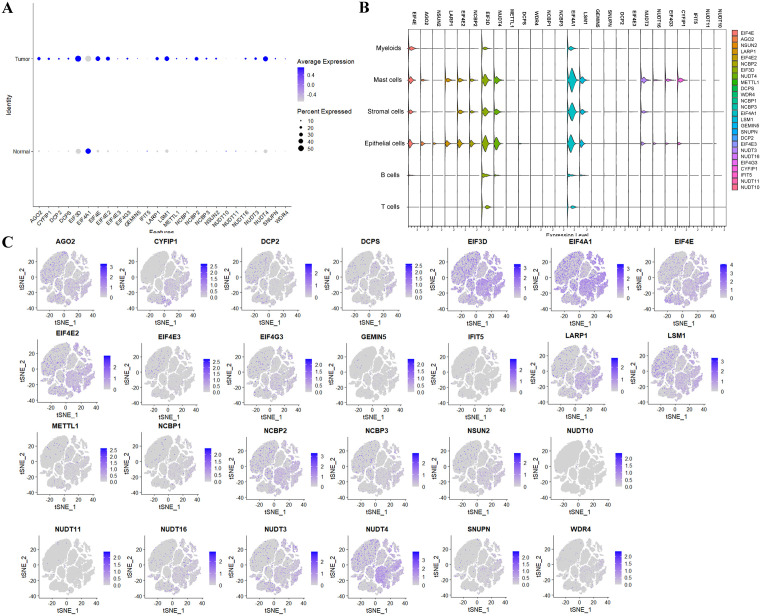
m7G-related Gene Expression in Single-Cell Samples. **A)** Bubble plots of the m7G-related gene expression **B)** Violin plots of the m7G-related gene expression. **C)** t-SNE plots of the m7G-related gene.

After reviewing the related literature and identifying 26 m7G-related genes, we used heatmap functions to observe the expression of these genes across various samples. In Fig 2A, it is evident that most genes were significantly more highly expressed in tumor tissues than in normal tissues, suggesting that m7G-related genes may promote tumor development. EIF3D and NUDT4 were highly expressed in normal tissues compared to other m7G-related genes. Additionally, we used Violin plot to show the average expression of m7G-related genes in each cell type, indicating that EIF4E, EIF3D, NUDT4, EIF4A1 and LSM1 were relatively highly expressed, mainly in the epithelial, mast, and stromal cells (Fig 2B). Using t-SNE plots, we displayed the distribution of m7G-related genes across various cell types, with NCBP2, EIF3D, and NUDT4 being predominantly expressed in epithelial cells. Most tumor cells expressed NCBP2, EIF3D, and NUDT4 suggesting that m7G-related genes may play a significant role in tumor development.

### m7G-related gene scoring and differential gene identification

**Fig 3:**
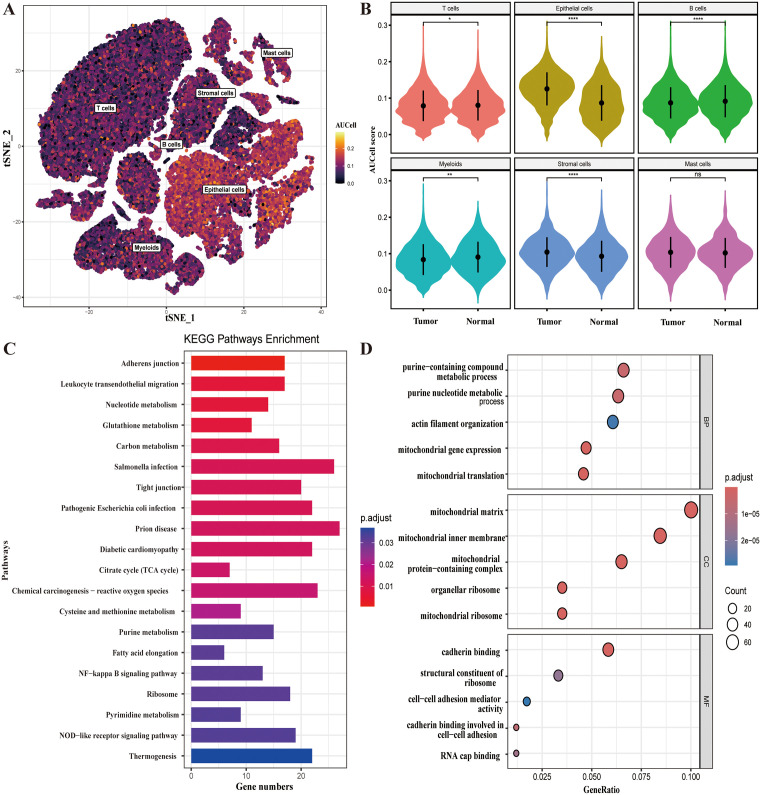
m7G-related Gene Scoring and Differential Gene Identification. **A)** t-SNE plot based on AUCell score. **B)** Violin plots of the AUCell score. **C)** Kyoto Encyclopedia of Genes and Genomes (KEGG) analysis of the DEGs. **D)** Gene ontology (GO) analysis of the DEGs.

With the AUCell R package’s scoring function and AddModuleScore, we calculated the average expression level score for 26 m7G-related genes. AUCell scores were visualized on a t-SNE plot. As seen in Fig 3A, epithelial cells scored the highest, indicating that m7G-related genes had the highest average expression and possibly the highest degree of m7G-related methylation. Additionally, we compared the AUCell scores between normal and tumor tissues. In Fig 3B, it is evident that epithelial and stromal cells in tumor tissues have higher average expression scores for m7G-related genes than those in normal tissues and show variability. The scores for T cells, B cells, and myeloid cells in tumor tissues were lower than those in normal tissues. There is evidence that m7G-related genes are expressed more highly in tumor tissues than in normal tissues, and these genes are involved in the development and growth of CRC tumors and stromal cells. T cells, B cells, and myeloid cells expressed less m7G-related genes in tumor tissues, which may be due to the tumor’s immunosuppressive microenvironment. For differential expression analysis, all cells were divided into high- and low-expression groups according to expression scores. Genes with a log2FC absolute value > 1 and p.adj < 0.05 were subjected to KEGG and GO analysis (Fig 3C, D). Differential genes were involved in pathways such as Adherens junction, Leukocyte transendothelial migration, Nucleotide metabolism, Glutathione metabolism, Carbon metabolism, Tight junction, and Chemical carcinogenesis – reactive oxygen species,etc. This shows that m7G modifications are related to inflammatory signaling pathways, cell adhesion, and metabolism and may be involved in tumor cell proliferation and metastasis. Additionally, GO analysis revealed enrichment in Biological Process terms, including purine-containing compound metabolic process, purine nucleotide metabolic process, actin filament organization, mitochondrial gene expression, and mitochondrial translation. The Cellular Component terms primarily involved mitochondria and ribosomes. Molecular Function terms focused on cadherin binding, structural constituent of ribosome, cell-cell adhesion mediator activity, cadherin binding involved in cell-cell adhesion, and RNA cap binding. This suggests that m7G-related genes are closely associated with ribosomes and mitochondria and participate in cell adhesion and transcriptome modification.

### Cell communication analysis (CellChat)

**Fig 4:**
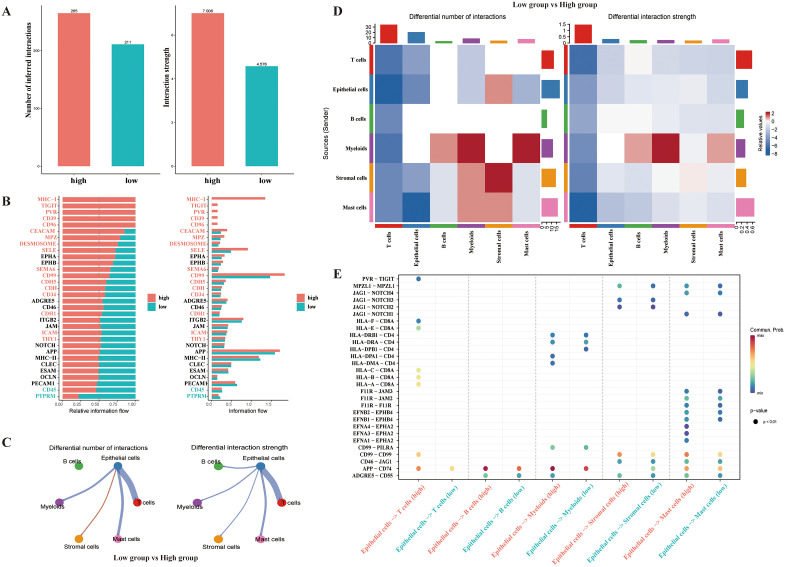
Cell Communication Analysis. **A)** The number and strength of interactions in different groups. **B)** The information flow in different groups. **C)** The circle of the number and strength of interactions. **D)** Heatmap of interaction numbers (left) and interaction strength (right). **E)** The bubble diagram of ligand-receptor mediated cell interactions.

We conducted a cell communication analysis of tumor sample-derived cells using CellChat. According to median AUCell scores, patients were divided into high- and low-scoring groups with varying average expression levels of m7G-related genes. In Fig 4A, the high-scoring group showed a greater number and intensity of cell communications than the low-scoring group. As illustrated in Fig 4B, signals for MHC-1, TIGIT, PVR, CD39, CD96, CEACAM, MPZ, DESMOSOME, SELE, SEMA6, CD99, CDH5, CDH, CDH1, ICAM, and THY were stronger in the high-scoring group than in the low-scoring group. The CD99 signal was the most significant in the high-scoring group, surpassing that in the low-scoring group. Furthermore, we analyzed the differences in communication between epithelial cells and other cells. In the high-scoring groups, epithelial cells exhibited more communication with myeloid cells, stromal cells, T cells, and mast cells compared to the low-scoring group, with no difference in communication with B cells. In terms of intensity, communications from epithelial cells to all other subgroups were stronger in the high-scoring group. This indicated that high m7G-related gene set scores enhanced epithelial cell interactions with other cells. We also compared the communication strengths and quantities among all the cells. Overall, interactions among cells in the high-expression group were more abundant than those in the low-expression group, with the main differences between epithelial and T cells in terms of quantity and intensity. The strength of the myeloid and stromal cells was higher in the low-scoring group than in the high-scoring group. Next, we compared the ligand-receptor interactions between epithelial cells and other cells in different groups. In the high-scoring group, interactions between epithelial cells and T cells, B cells, and myeloid cells, particularly APP-CD74 interactions, were stronger than those in the low-scoring group. Interactions between epithelial cells and T cells, such as PVR-TIGIT, HLA-CD8A, and CD99-CD99, were stronger in the high-scoring group than those in the low-scoring group.

### Analysis of epithelial cells

**Fig 5:**
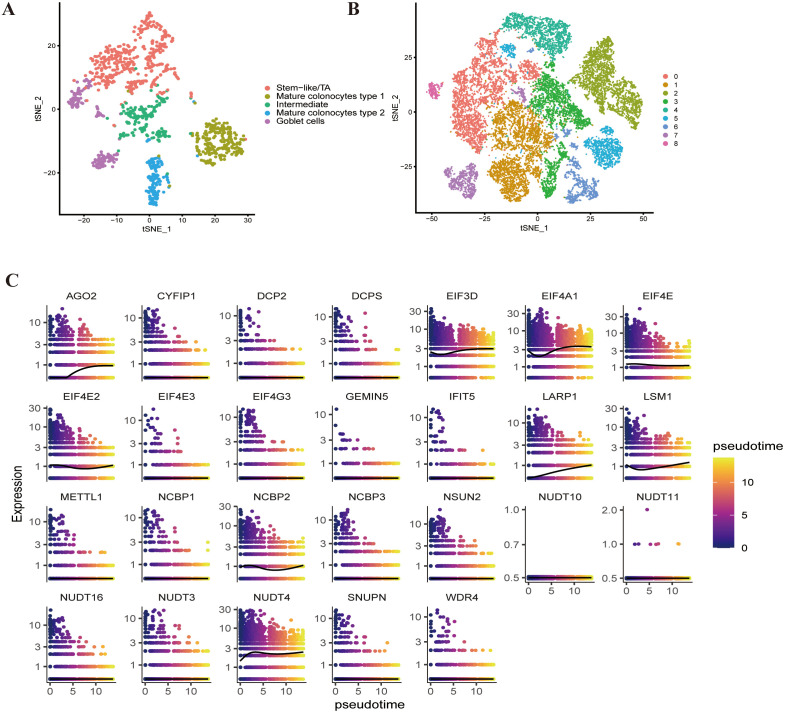
Analysis of Epithelial Cells. **A)** t-SNE plot of epithelial cells of normal tissue. **B)** t-SNE plot of epithelial cells of tumor tissue. **C)** Pseudo-time trajectory of m7G-related genes.

Based on previous cell clustering, we isolated epithelial cells originating from normal tissue samples and categorized them according to standard markers for each cell group reported in the literature [46]. The epithelial cells were classified into five types: stem-like cells, colonocytes type 1, intermediate colonocytes type 2, and goblet cells (Fig 5A) (S2 Fig). Clustering of epithelial cells derived from tumor tissues resulted in nine groups, and CNV analysis was performed to assess the malignancy level, identifying all nine subgroups as malignant (S3 Fig). Subsequently, we used the monocle3 R package for pseudo-time analysis of malignant epithelial cells, simulating differentiation into specific subgroups to observe whether m7G-related genes participate in the differentiation process of malignant tumor cells. Clustering of malignant epithelial cells using UMAP identified 15 subgroups, indicating that nine m7G-related genes (AGO2, EIF3D, EIF4A1, EIF4E, EIF4E2, LARP1, LSM1, NCBP2, and NUDT4) are involved in the differentiation of malignant tumor cells, with gene expression continuously changing during the transition process (Fig 5C).

### Analysis of T Cells, B cells, and metabolism

**Fig 6:**
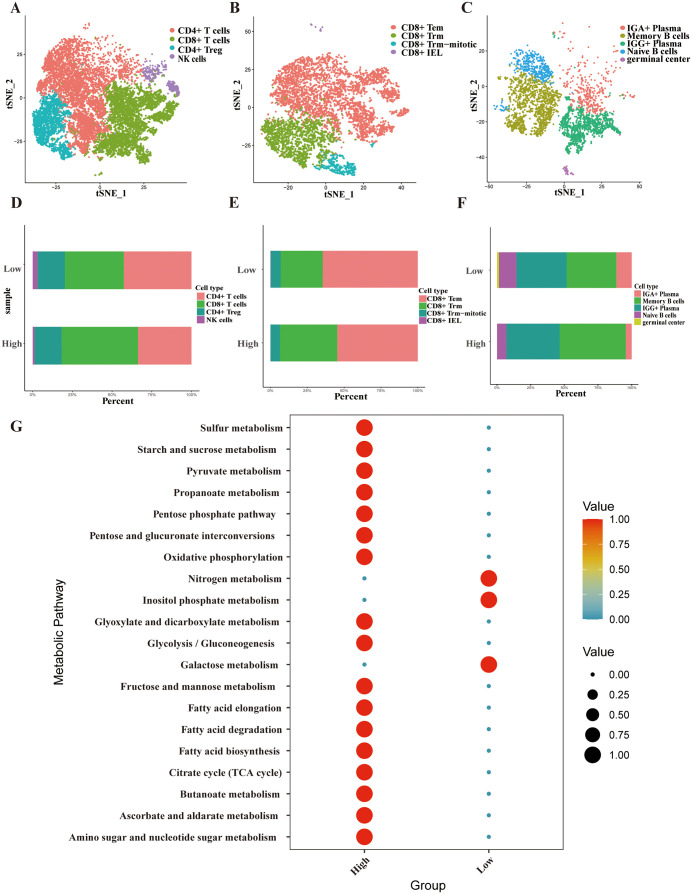
Analysis of T Cells, B Cells, and Metabolism. **A)** t-SNE plot of T cells. **B)** t-SNE plot of CD8 + T cells. **C)** t-SNE plot of B cells. **D)** Cell proportion of T cells. **E)** Cell proportion of CD8 + T cells. **F)** Cell proportion of B cells. **G)** The bubble diagram of metabolism pathway scores.

We also analyzed the impact of m7G modifications on the two main types of immune cells, T and B cells, from a single-cell perspective. T cells from tumor tissues were extracted and annotated into four types (CD4 + T cells, CD8 + T cells, CD4 + Tregs, and NK T cells) based on Cell Mark 2.0 and literature references (Fig 6A) [47]. The t-SNE plots shows representative markers of several cell types, with CD4+ and CD8 + cells being the most abundant. The high-scoring patients had a higher proportion of CD8 + T cells, whereas the low-scoring patients had more CD4+ and NK T cells. Based on the literature, CD8 + T cells were further subdivided into four groups: CD8 + Tem, CD8 + Trm, CD8 + Trm-mitotic, and CD8 + IEL (Fig 6B) (S4 Fig). High-scoring patients had a higher proportion of CD8 + Trm cells, whereas CD8 + Tem cells were less common than in low-scoring patients. CD20(MS4A1)+ B cells and plasma B cells (S5 Fig) were separated; plasma cells were further divided into Memory B cells and IGG+ Plasma; CD20 + B cells were further divided into three types (IGA+ Plasma, Naive B cells, and germinal center) (Fig 6C). High-scoring patients had more CD8 + Trm and fewer CD8 + Tem cells than low-scoring patients. Through the analysis of the two main types of immune cells, T and B cells, we discovered that m7G modifications affected both, reducing the proportion of tumor immune cells.

A metabolic pathway enrichment analysis was conducted for high- and low-scoring patient groups to investigate the effects of m7G modification on tumor cell metabolism. The top 20 pathways with the most significant differences were analyzed using the scMetabolism R package. The results indicated that the high-scoring group had a higher enrichment of tumor cell metabolic pathways than the low-scoring group, including major metabolic pathways (Fatty acid elongation, Fatty acid degradation, Fatty acid biosynthesis, Citrate cycle (TCA cycle), Butanoate metabolism, Ascorbate and aldarate metabolism, and Amino sugar and nucleotide sugar metabolism). Nitrogen metabolism, Inositol phosphate metabolism, and Galactose metabolism were more active in the low-scoring group than in the high-scoring group. Thus, m7G modification also affects tumor cell metabolism, potentially enhancing tumor cell energy metabolism, fat metabolism, and amino acid metabolism.

### NMF clustering analysis

**Fig 7:**
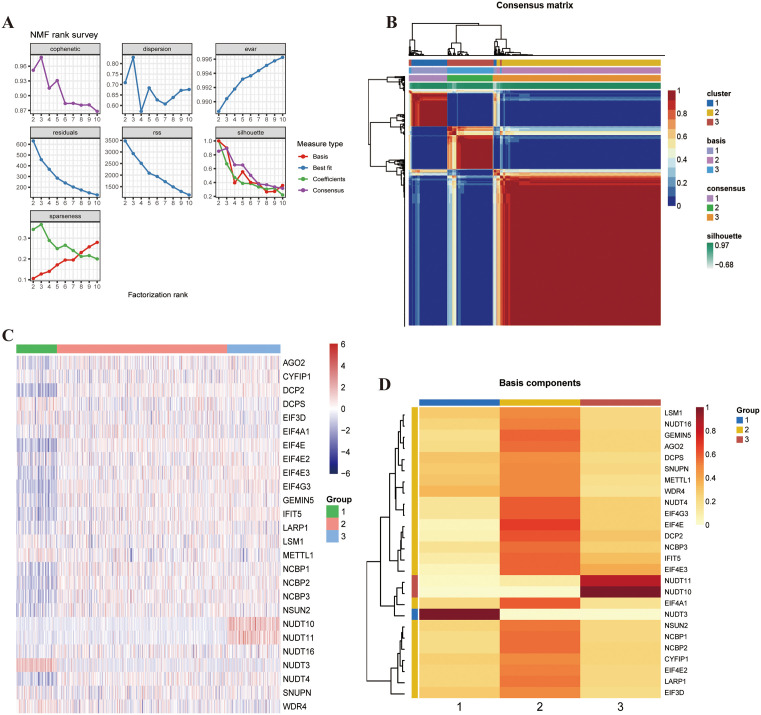
NMF Clustering Analysis. **A)** NMF rank survey. **B)** Heatmap of clustering at consensus k = 3. **C, D)** Heatmap of m7G-related gene expression in three clusters.

In addition to single-cell datasets, we explored the m7G-related gene set using bulk transcriptome sequencing. We combined and processed the data from COAD and READ patients in the TCGA database. Patients with prognostic information were selected, and those with a survival time of 0 were removed, resulting in data from n patients. Using NMF unsupervised clustering analysis, we clustered the expression of 26 m7G-related genes from 591 patients in the TCGA database. By selecting a rank value of 3 when cophenetic descent was the fastest, we divided patients with different features of m7G-related genes into three clusters (Fig 7A, B). In the subsequent heatmaps, A total of 26 m7G-related genes were detected in different clusters. The second group of patients had specific high expression levels of two genes (NUDT10 and NUDT11), the third group had specific high expression levels of one gene (NUDT3), and the first group had relatively high expression levels of the remaining 23 genes (Fig 7C, D).

### Immune correlation study

**Fig 8:**
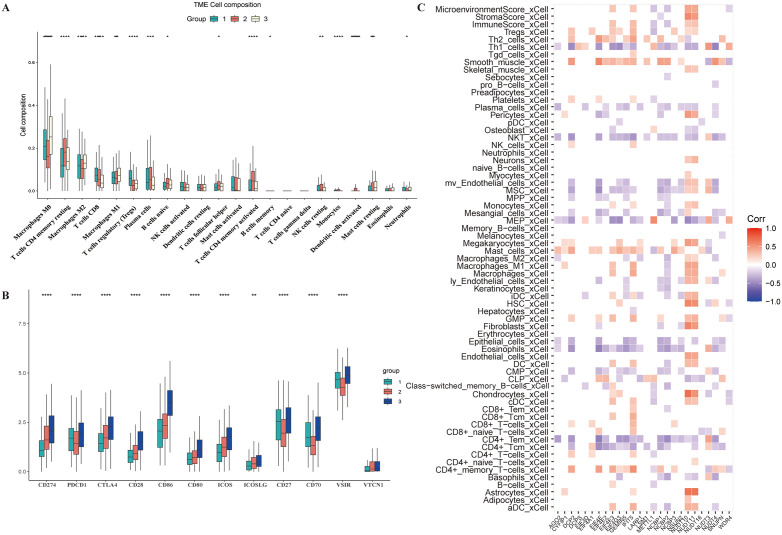
Immune Correlation Study. **A)** TME cell composition of three clusters. **B)** The expression of immune checkpoint related genes in three clusters. **C)** The correlation analysis between immune cells and m7G-related gene expression.

To find the differences among the three groups based on m7G-related gene grouping, we analyzed survival differences. However, no differences were found among the three groups. Subsequently, using the CIBERSORT R package, we compared immune infiltration between the different feature groups, revealing many differences in immune cells among the three groups. In the second group, M0 macrophages, M2 macrophages, M1 macrophages, and resting mastcells were more abundant than in the other two groups. T cells CD4 memory resting, T cells CD4 memory activated, and T cells follicular helper were most prominent in the first group. CD8 T cells and plasma cells were highest in the third group and lowest in the second group (Fig 8A). The expression of immune checkpoint genes was then analyzed across the three groups, with the highest expression observed in the second group, showing significant differences in immune checkpoint genes, such as CD274, PDCD1, and CTLA4. These results suggest varying roles for different m7G-related genes in tumor immune regulation (Fig 8B). Furthermore, using Xcell, we analyzed the expression characteristics of 64 immune cells and stromal cells and correlated them with m7G-related genes. In Fig 8C, m7G-related genes showed correlations with immune cells, with p-values less than 0.05 displayed in a red or blue module. Most genes negatively correlated with the immune system, whereas NUDT10 and NUDT11 positively correlated with most immune cells. CD4 + Tem and NK T cells negatively correlated with almost all m7G-related genes. NCBP2 expression negatively correlated with CD8 + Tem cells, M1 macrophages, and M2 macrophages. In terms of the overall microenvironment and immune score, METTL1 and NCBP2 negatively correlated with the immune score and showed significant differences. By comparing 64 immune cells with m7G-related genes, we confirmed the involvement of m7G in the immune regulation of colorectal tumors.

### Selection of hub genes and construction of prognostic models

**Fig 9:**
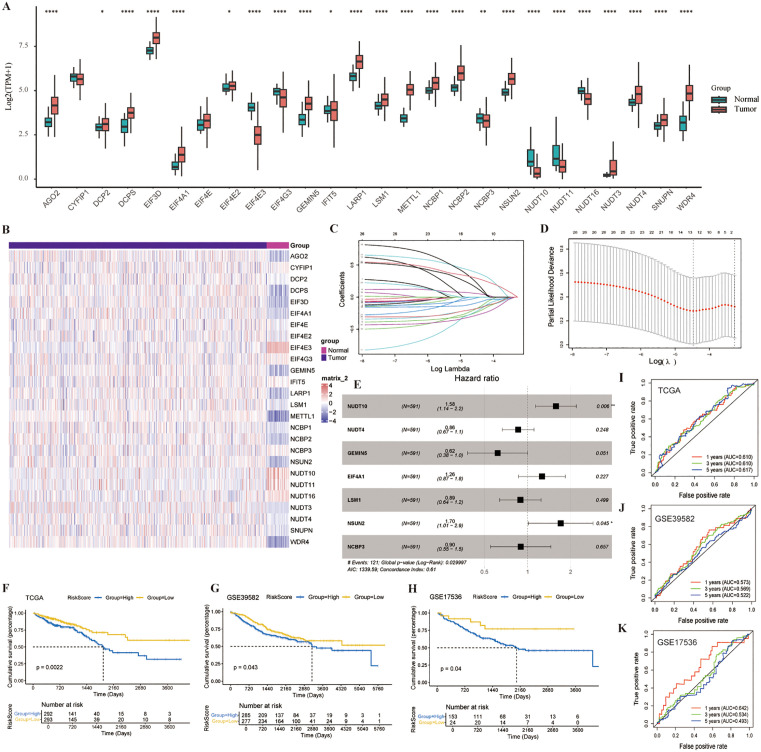
Selection of Hub Genes and Construction of Prognostic Models. **A)** m7G-related gene expression in normal and tumor groups. **B)** Heatmap of m7G-related gene expression in normal and tumor groups. **C, D)** LASSO logistics regression and the optimal parameter λ. **E)** Regression model. **F, G, H)** The Curve of survival in TCGA, GSE39582 and GSE17536 cohorts. **I, J, K)** A time-dependent ROC curve in TCGA, GSE39582 and GSE17536 cohorts.

TCGA-COAD and TCGA-READ were used to compare expression levels of 26 m7G-related genes. In Fig 9A, we observed that most genes were upregulated in tumor tissues, which agree with the findings from the study of a previous single-cell dataset. Only EIF4E3, IFIT5, EIF4G3, NUDT16, NUDT10, NCBP3, and NUDT11 showed decreased expression in tumor tissues. Genes such as CYFIP1 and EIF4E exhibited no differences in expression between the two groups. Subsequently, a heatmap was utilized for a more intuitive display, showing similar results to the previous figure, with EIF4E3, EIF4G3, NUDT16, NUDT10, NCBP3, and NUDT11 displaying increased expression (shown in red) in normal tissues, whereas the rest of the genes showed decreased expression (shown in blue). Clinical information from all patients was extracted from TCGA, and LASSO regression was employed to narrow down the candidate gene range, leading to the identification of 13 key genes. m7G-related genes were screened using univariate Cox regression analysis and the optimal C-index, ultimately selecting the seven most significant genes: NUDT10, NUDT4, GEMIN5,EIF4A1,LSM1,NSUN2, and NCBP3(Fig 9E). Ultimately, those genes were retained to construct the most suitable prognostic prediction model. NUDT10 showed a positive correlation with death events, with a coefficient of 1.58, and NSUN2 showed a positive correlation with death events, with a coefficient of 1.70 (Fig 9E). Subsequently, a risk score calculation was performed on their expression according to the equation: risk score = NUDT10 × 0.4559 - NUDT4 × 0.1506- GEMIN5 × 0.4804 + EIF4A1 × 0.2323- LSM1 × 0.1149 + NSUN2 × 0.5321- NCBP3 × 0.1097. The C-index values of our predictive model were 0.609, 0.587, and 0.659 in the TCGA, GSE39582, and GSE17536 datasets, respectively. These results are comparable to those reported in previous studies by Zheng *et al*. (0.544, 0.596, 0.603), Du *et al*. (0.621, 0.605, 0.632), and Liang *et al*. (0.678, 0.601, 0.658) [48–50]. Decision curve analysis (DCA) was employed to evaluate the clinical utility of the prognostic model. As illustrated in S6 Fig, the standardized net benefit of the models derived from the TCGA, GSE39582, and GSE17536 datasets were compared against two default strategies: intervening for all patients (“All”) or for no patients (“None”). The DCA curves demonstrated that the application of our model yielded a higher net benefit across a wide range of high-risk thresholds (approximately 0% to 40%) compared to the simple “All” or “None” strategies. Specifically, the models based on the TCGA (blue curve) and GSE39582 (red curve) cohorts showed superior and very similar net benefit, particularly within the lower threshold probability range. The GSE17536 (light blue curve) model also provided a positive net benefit, although it was slightly lower than that of the other two models across most thresholds. Following this, each patient in the TCGA database was assigned a risk score, and survival differences were compared between the high-score and low-score groups. Differences in the Kaplan–Meier curves between the high- and low-score groups were notable (P = 0.0022), with the low-score group demonstrating a better prognosis than the high-score group (Fig 9G). The AUC values in the ROC curve were 0.610 at 1 year, 0.610 at 3 years, and 0.617 at 5 years, falling within the range of 0.5–0.7, indicating a moderate predictive performance (Fig 9I). Validation was conducted using the GSE39582 dataset from the GEO database, with results showing a better prognosis in the low-score group than in the high-score group used the optimal cutoff point to divided patients into the high- and low-risk groups, as demonstrated by differences in the Kaplan–Meier curves (P = 0.043) (Fig 9H). ROC curve AUC values were 0.573 at 1 year, 0.569 at 3 years, and 0.522 at 5 years (Fig 9J). The GSE17536 dataset from the GEO database also showed a better prognosis in the low-score group than in the high-score group that used the optimal cutoff point to divide patients into the high- and low-risk groups (P = 0.04). ROC curve AUC values were 0.642 at 1 year, 0.534 at 3 years, and 0.493 at 5 years (Fig 9K).

### RNA Sequencing (RNA-Seq) and Immunohistochemistry (IHC)

**Fig 10:**
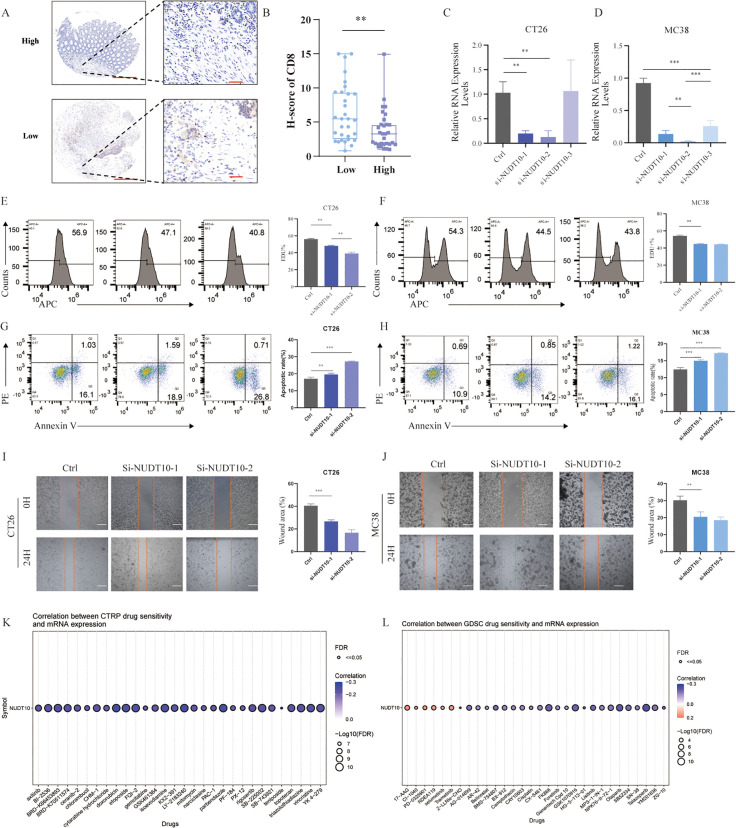
NUDT10 promotes the progression of CRC. **A, B)** immunohistochemistry and score. **C, D)** qPCR of CT26 and MC38. **E, F)** EdU of of CT26 and MC38. **G, H)** Apoptosis of of CT26 and MC38. **I, J)** Wound healing test of CT26 and MC38. **K, L)** Correlation between drug sensitivity and mRNA expression.

RNA-Seq was performed on tumor tissues from 44 patients. Based on the expression levels of two hub genes, a risk score was calculated using a predefined formula. Patients were then stratified into high- and low-risk groups. Immunohistochemistry (IHC) was employed to evaluate CD8 + T cell infiltration within the tumor microenvironment. The results demonstrated that the low-risk group exhibited significantly higher CD8 + T cell infiltration, whereas the high-risk group showed markedly reduced infiltration (p < 0.05). Furthermore, the high-risk group displayed characteristics of an immunosuppressive tumor microenvironment compared to the low-risk group (Fig 10A,B).

### NUDT10 promotes the progression of CRC

We selected NUDT10, a gene that has been relatively understudied in predictive models, to investigate its functional role in colorectal cancer. Using siRNA-mediated knockdown, we achieved efficient gene silencing and determined that si-NUDT10–2 exhibited the highest interference efficiency (Fig 10C,D). Consequently, both si-NUDT10–1 and si-NUDT10–2 were selected for subsequent functional experiments. In both CT26 and MC38 cell lines, the knockdown groups exhibited a decreased proportion of proliferating cells and an increased rate of apoptosis, indicating that inhibition of NUDT10 can suppress the progression of cancer cells. Subsequently, the scratch test further demonstrated that inhibition of NUDT10 impeded the migration of tumor cells.

Based on the pharmacogenomic analysis from the CTRP and GDSC databases, we investigated the correlation between NUDT10 mRNA expression and the sensitivity of cancer cells to a broad spectrum of anticancer compounds (Fig 10K,L). The CTRP analysis revealed that elevated NUDT10 expression was significantly associated with increased resistance to 26 specific drugs (Correlation < 0, FDR < 0.05). These agents include Axitinib (VEGFR inhibitor), BI-2536 (PLK1 inhibitor), suggesting a potential role for NUDT10 in mediating chemoresistance. The GDSC analysis further corroborated this trend, showing positive correlations with sensitivity to HSP90 inhibitor (17-AAG), MEK inhibitors (e.g., CI1040, PD0325901, Selumetinib, Trametinib), indicating that high NUDT10 expression was associated with greater sensitivity to specific targeted agents.

## Discussion

Currently, the main treatment for CRC is surgery with adjuvant chemotherapy; however, there remains a risk of drug resistance and metastasis. Targeted therapies are only effective for specific molecular subtypes, and immunotherapy is only effective in approximately 10% of patients with d-MMR/MSI-H. Therefore, CRC development and occurrence require in-depth studies of regulatory mechanisms.

In recent years, m7G modification has received significant attention as a type of RNA modification. The targets of m7G modification are diverse, including tRNA, miRNA, and mRNA, ultimately affecting gene expression [51]. Like m6A, m7G-related genes are divided into three core regulatory factors: writer, reader, and eraser. m6A acts as a “precision control switch” for mRNA metabolism, participating in nearly all its regulatory processes, whereas m7G (particularly the 5’ cap structure) functions more like an “ignition key” for mRNA. While the role of m6A in tumors has been extensively studied, the functional significance of m7G modification remains unclear. Owing to certain limitations in the currently available research, more studies are needed to identify m7G-related genes.

We investigated the relationship between m7G modification and CRC at the single-cell level in this study. Our study examined the relationship between CRC immune infiltration and m7G modification to understand the impact of m7G modification on the immune microenvironment and to identify potential therapeutic targets and prognostic predictors. We used the publicly available CRC single-cell dataset from GEO and performed dimensionality reduction clustering on all cells. The cells were classified into six subgroups: T cells, epithelial cells, B cells, myeloid cells, stromal cells, and mast cells. The proportion of epithelial cells in tumor tissues was significantly higher than in normal tissues, whereas T cells and B cells decreased, indicating an immunosuppressive microenvironment. Subsequently, we visualized the expression of the 26 m7G-related genes using heatmaps and t-SNE plots. In tumor tissues, most genes expressed at higher levels than in normal tissues, primarily within epithelial cells. This is consistent with previous findings that m7G genes are highly expressed in tumors, promoting the occurrence and development of malignant epithelial cells. Using AUCell, we scored all m7G gene sets and found that epithelial cells had the highest scores, with epithelial cell and stromal cell scores in tumor tissues being higher than those in normal tissues. Differential gene enrichment analysis using KEGG and GO revealed that differentially expressed genes tended to be enriched in pathways related to inflammation and metabolism. Cell-cell communication analysis using CellChat showed that patients with high m7G-related gene expression had stronger and more frequent cell communication between epithelial cells and other cells than patients with low m7G-related gene expression. Additionally, patients with a high expression of m7G-related genes showed stronger interactions with certain receptors, potentially indicating a mechanism by which m7G modification promotes tumor development and suggesting that inhibiting these pathways could suppress tumor growth. Through pseudo-time analysis, we identified genes such as NCBP2, EIF3D, and NUDT4 boost the occurrence and development of malignant tumor epithelial cells, providing guidance for identifying potential therapeutic targets for CRC. Additionally, high-score patients had a different immune microenvironment from low-score patients. CRC patients with a better prognosis had a higher proportion of CD8 + T cells and a lower proportion of CD4 + T cells than patients with a worse prognosis.

Our study also revealed that m7G affects tumor metabolism. In metabolic pathways, the high-expression group showed higher levels than the low-expression group, except for Nitrogen metabolism, Inositol phosphate metabolism, and Galactose metabolism.

We validated the effects of m7G-related genes on CRC using bulk sequencing. Using NMF, we classified patients with m7G-related features into three types, among which differences in the expression levels of m7G-related genes were observed. Although patient survival did not differ between subtypes, there were significant differences in the immune environment. The expression of immune checkpoint markers varied among the different subtypes, suggesting that differences in the m7G modification status may affect the response to immunotherapy. Subsequently, we studied the correlation of each m7G-related gene with immune cells and found that these genes had varying degrees of impact on immune cells. Genes such as EIF4E3, IFIT5, EIF4G3, NUDT16, NUDT10, NCBP3, and NUDT11 showed lower expression in tumor tissues, whereas other genes were highly expressed.

We used LASSO Cox regression analysis to select 13 key prognostic genes (DCPS, EIF3D, EIF4A1, EIF4E3, GEMIN5, METTL1, NCBP1, NCBP2, NSUN2, NUDT4, NUDT10, SNUPN, and WDR4) among the m7G-related genes. Seven genes (NUDT10, NUDT4, GEMIN5,EIF4A1,LSM1,NSUN2, and NCBP3) were selected for prognostic model construction and validated using the GEO dataset GSE39582 and GSE17536. NUDT10 and NSUN2 are two genes significantly negatively correlated with patient prognosis. Among them, NSUN2 has been extensively reported to be significantly highly expressed in colorectal cancer (CRC) and is closely associated with glycolysis. It promotes the occurrence and metastasis of colorectal cancer by maintaining mRNA stability.In contrast, research on NUDT10 is relatively limited. NUDT10 belongs to the nudix (nucleoside diphosphate-linked moiety X) family. NUDT10 showed lower expression in tumor tissues than in normal tissues, and patients with high expression in tumor tissues had lower survival rates than those with low expression, consistent with similar reports(e.g., lung squamous cell carcinoma, Gastric Cancer, Clear Cell Renal Cell Carcinoma, Hepatocellular Carcinoma) in the literature [52–54].Subsequently, we conducted relevant biological experiments that confirmed its tumor-promoting function in CRC.

It is important to note that this research had certain limitations, including a limited number of single-cell samples, the need for more in-depth research, differences in sequencing methods and treatment modalities in bulk sequencing analysis, and the limited predictive value of the prognostic model. Nevertheless, our study identified major subgroups of m7G modifications occurring at the single-cell level. Moreover, we explored genes affecting the development of malignant epithelial cells and elucidated complex interactions from immune and metabolic perspectives. We also conducted a comparison of immune environments in three patient groups with different characteristics using CRC bulk RNA-seq to identify key prognostic factors.

## Conclusions

For the first time, the expression of genes associated with m7G modification was examined from a single cell sequencing perspective, revealing tumor growth and microenvironment changes mediated by m7G modification. We subtyped m7G modification in bulk transcriptomes to investigate its impact on immune function. We built a model for prognosis based on seven hub genes, which will guide the further exploration of m7G modifications. Furthermore, the study elucidated the role of NUDT10 in promoting colorectal tumor progression.

## Supporting information

S1 Fig Bubble plots of markers of different cell types. (TIFF)

S2 Fig Bubble plots of different epithelial cells. (TIFF)

S3 Fig InferCNV of different epithelial cells. (PNG)

S4 Fig Bubble plots of different CD8 T-cells. (TIFF)

S5 Fig MS4A1 expression distribution. (TIFF)

S6 Fig DCA of different datasets. (TIF)

S1 Table RNA-seq data. (CSV)
