# Supplementary figures and images for: Analyses of the roles and potential targets of m7G-related genes in colorectal cancer using single-cell and bulk RNA sequencing data
Source: PLoS One. 2025 Dec 17;20(12):e0337288. doi: 10.1371/journal.pone.0337288 (PMC12711009; doi:10.1371/journal.pone.0337288)

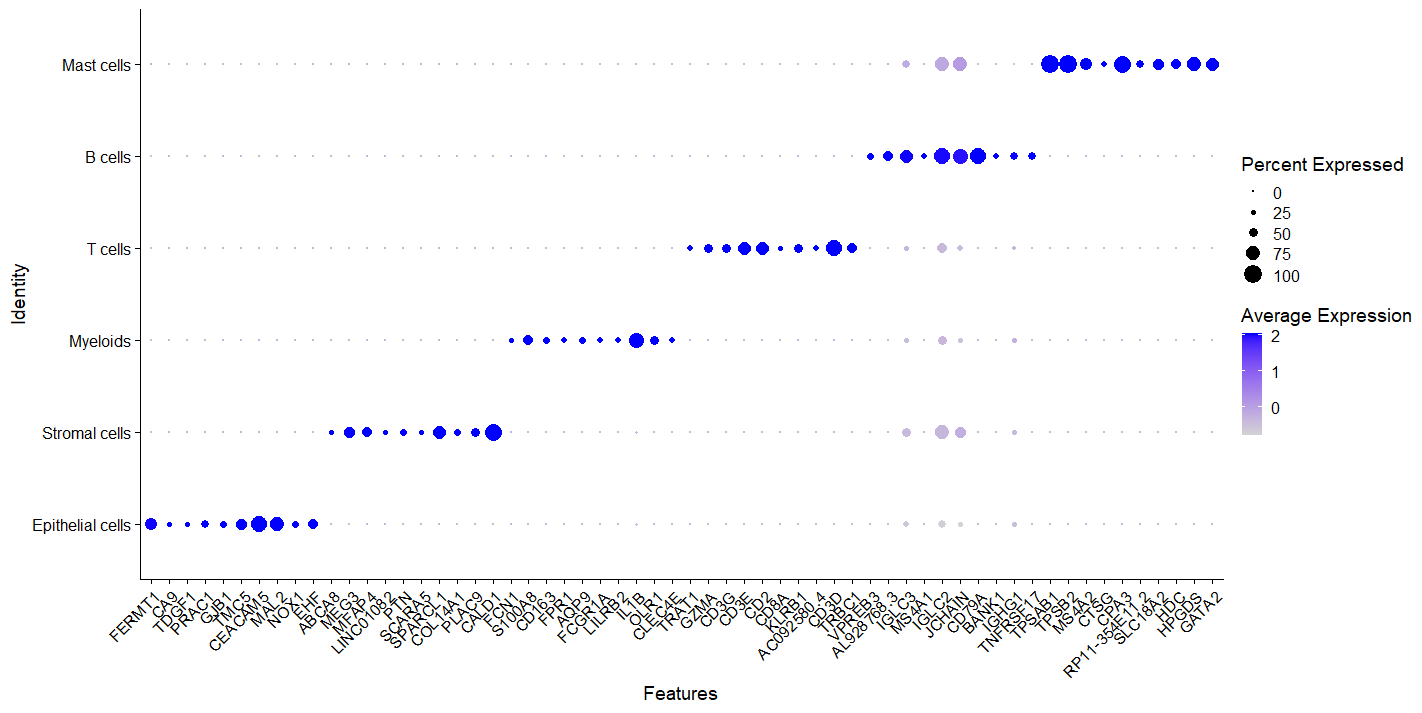

Supplement: S1 Fig — (TIFF) [file pone.0337288.s001.tiff]

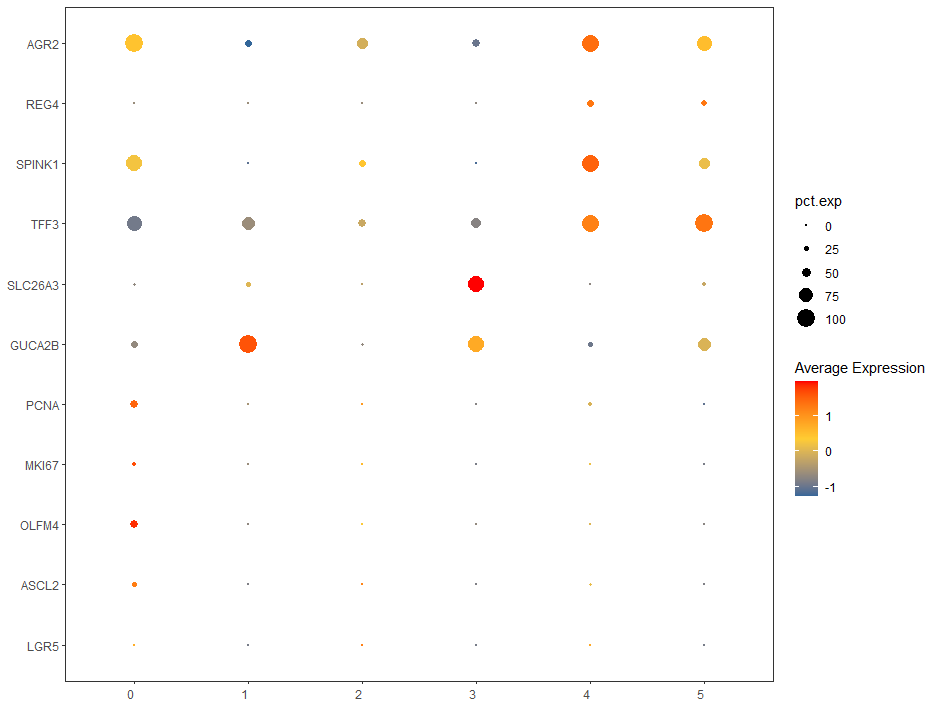

Supplement: S2 Fig — (TIFF) [file pone.0337288.s002.tiff]

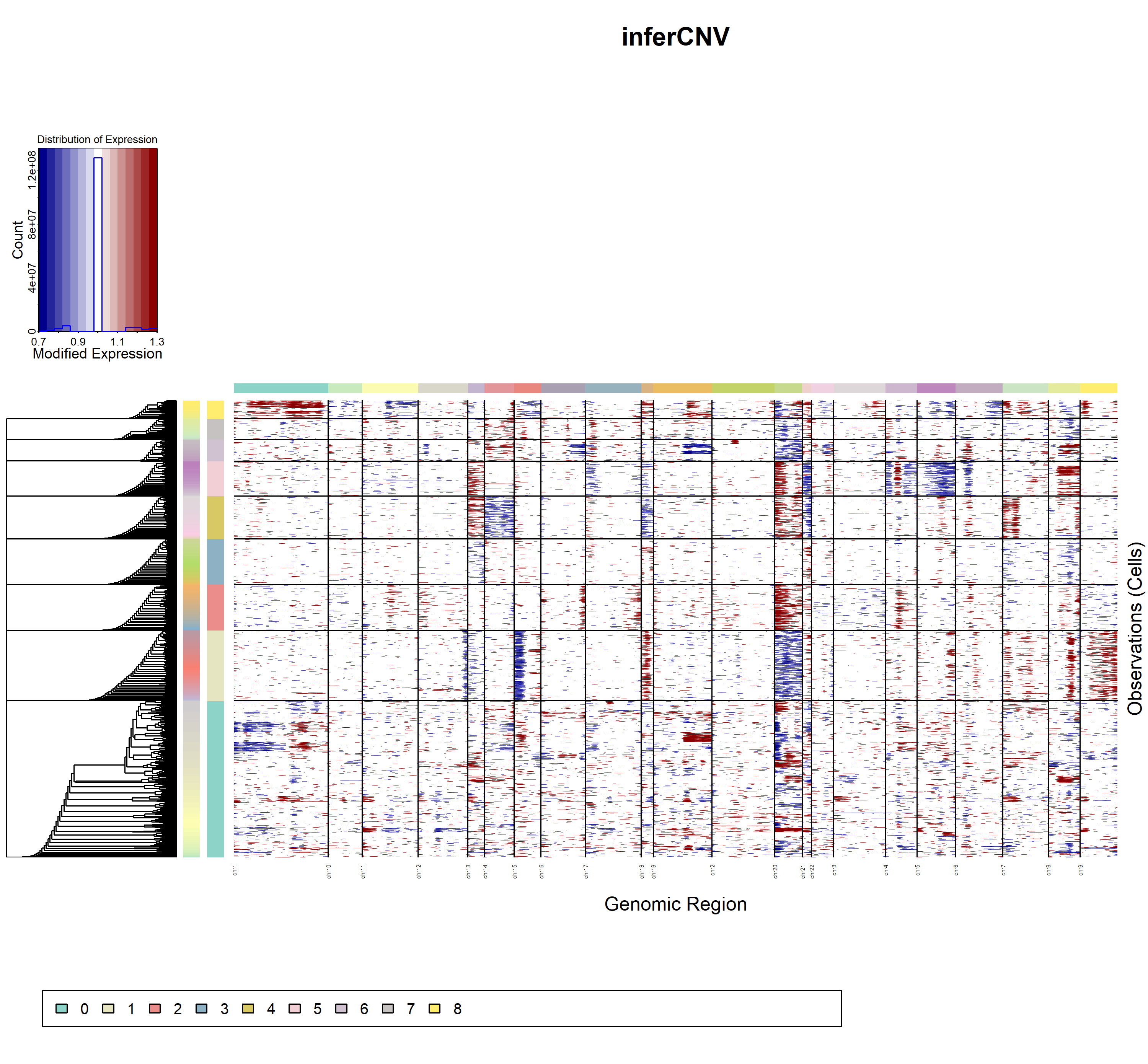

Supplement: S3 Fig — (PNG) [file pone.0337288.s003.png]

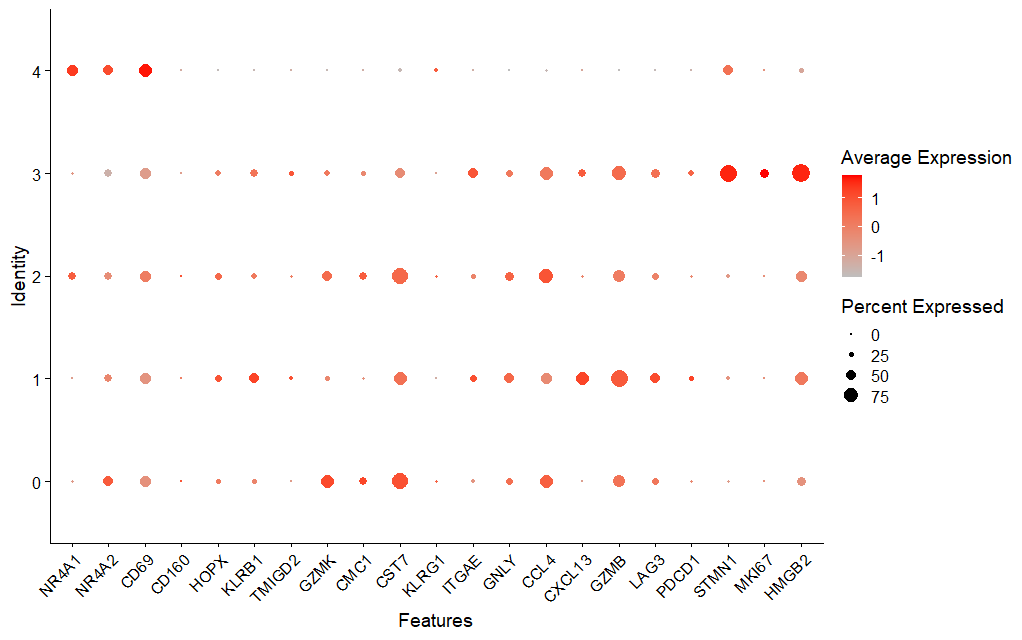

Supplement: S4 Fig — (TIFF) [file pone.0337288.s004.tiff]

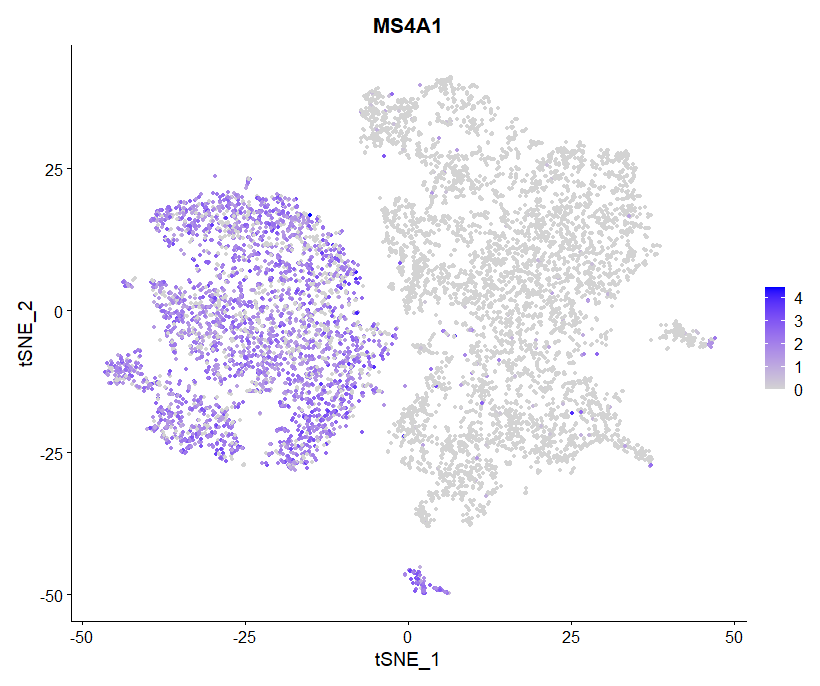

Supplement: S5 Fig — (TIFF) [file pone.0337288.s005.tiff]

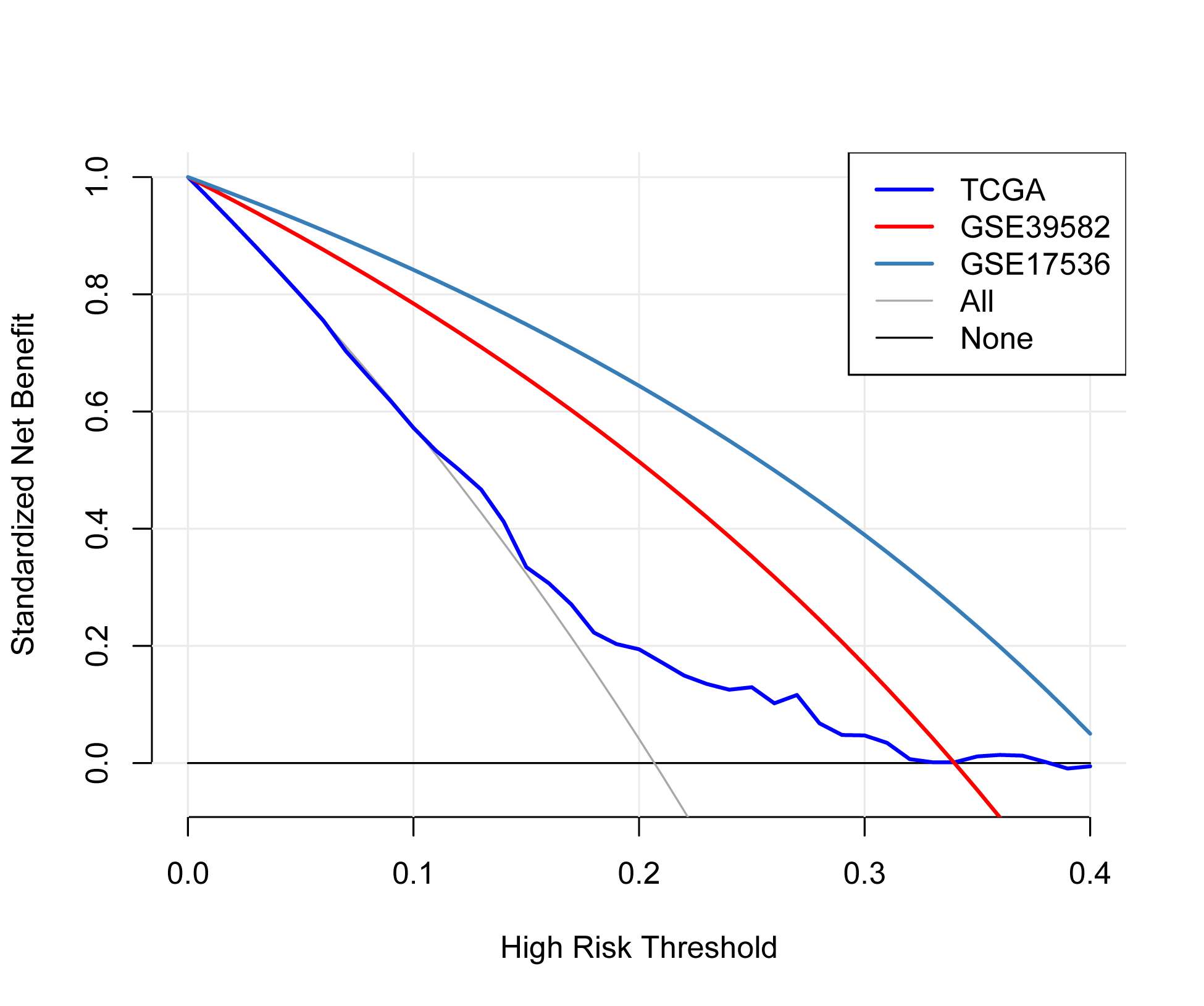

Supplement: S6 Fig — (TIF) [file pone.0337288.s006.tif]
